# Decoding mitochondrial heterogeneity in single muscle fibres by imaging mass cytometry

**DOI:** 10.1038/s41598-020-70885-3

**Published:** 2020-09-18

**Authors:** Charlotte Warren, David McDonald, Roderick Capaldi, David Deehan, Robert W. Taylor, Andrew Filby, Doug M. Turnbull, Conor Lawless, Amy E. Vincent

**Affiliations:** 1 Wellcome Centre for Mitochondrial Research, Translational and Clinical Research Institute, Newcastle University, Framlington Place, Newcastle upon Tyne, NE2 4HH UK; 2 Flow Cytometry Core Facility, Biosciences Institute, Newcastle University, Newcastle upon Tyne, NE2 4HH UK; 3 Cellstate Biosciences, Tuscon, AZ 85721 USA; 4 Translational and Clinical Research Institute, Newcastle University, Newcastle upon Tyne, NE2 4HH UK; 5 NHS Highly Specialised Rare Mitochondrial Disorders Service, Newcastle Upon Tyne Hospitals, NHS Foundation Trust, Newcastle upon Tyne, NE2 4HH UK

**Keywords:** Translational research, Cellular neuroscience, Molecular neuroscience, Mechanisms of disease

## Abstract

The study of skeletal muscle continues to support the accurate diagnosis of mitochondrial disease and remains important in delineating molecular disease mechanisms. The heterogeneous expression of oxidative phosphorylation proteins and resulting respiratory deficiency are both characteristic findings in mitochondrial disease, hence the rigorous assessment of these at a single cell level is incredibly powerful. Currently, the number of proteins that can be assessed in individual fibres from a single section by immunohistochemistry is limited but imaging mass cytometry (IMC) enables the quantification of further, discrete proteins in individual cells. We have developed a novel workflow and bespoke analysis for applying IMC in skeletal muscle biopsies from patients with genetically-characterised mitochondrial disease, investigating the distribution of nine mitochondrial proteins in thousands of single muscle fibres. Using a semi-automated analysis pipeline, we demonstrate the accurate quantification of protein levels using IMC, providing an accurate measure of oxidative phosphorylation deficiency for complexes I–V at the single cell level. We demonstrate signatures of oxidative phosphorylation deficiency for common mtDNA variants and nuclear-encoded complex I variants and a compensatory upregulation of unaffected oxidative phosphorylation components. This technique can now be universally applied to evaluate a wide range of skeletal muscle disorders and protein targets.

## Introduction

Investigation and analysis of muscle in ageing and disease is often complicated by the presence of both normal healthy fibres and those affected by pathology within a skeletal muscle biopsy. This is true of atrophy, senescence, degeneration, regeneration, inflammatory processes and necrosis among other pathological changes not to mention the heterogeneity of fibre-types^1,2^. Mitochondrial dysfunction is a particularly heterogeneous pathological change which occurs in sarcopenia, healthy ageing, primary mitochondrial disease and other neuromuscular disorders, such as inclusion body myositis^3^. Histological and immunohistochemical approaches allow investigation of protein expression and distribution at the single-cell and sub-cellular level and are therefore ideal for observing any mosaic pattern of affected and unaffected cells. However, multiple serial sections are often required to look at large numbers of markers, which is time consuming, uses large amounts of precious biopsy material and requires difficult tracing of fibres between serial sections.

Previously, immunofluorescent assays highlighted the contribution of mitochondrial oxidative phosphorylation deficiency to ageing and disease pathology^4–6^, fibre type switching and senescence in ageing^7^, allowed automated analysis of dystrophic changes in Duchenne muscular dystrophy^8^, allowed correlative analysis of protein aggregation^9^ and allowed the investigation of inflammatory processes^10^. However, a limitation of immunofluorescence (IF) and conventional immunohistochemistry (IHC) remains the relatively low number of protein targets that can be interrogated at a single cell level in a single tissue section due to spectral overlap of the fluorophores. Furthermore, the use of secondary antibodies together on a single cryosection is only possible if the primary antibody species and isotypes differ. Such limitations can be overcome by sequential staining of multiple antibodies on one tissue section, but this is time consuming and leads to a gradual degradation in tissue quality^11^.

The recent development of a high resolution laser ablation laser module has brought tissue imaging modality, at 1 μm per pixel resolution, to cytometry by time of flight mass cytometers (CyTOF). This imaging mass cytometry (IMC) technique utilises antibody-conjugated isotopes of rare earth metals and so is not limited by fluorophore associated spectral overlap. Thus, IMC allows for the simultaneous measurement of a greater number of proteins targets in a single section than is possible with standard immunofluorescent techniques^12^.

IMC also has important advantages compared to large-scale omics approaches. Proteomics has been used successfully to study oxidative phosphorylation deficiency in skeletal muscle homogenate and single cells^13^, however the abundance of cytoskeletal proteins can negatively impact results. Similarly, although transcriptomics has considerable potential, it would require the samples to have been collected and frozen rapidly which is challenging in a clinical setting. Perhaps the major advantage of IMC over large-scale omics approaches is that it allows subcellular spatial resolution of protein abundance. This will allow us to examine the cellular distribution of proteins which can provide important mechanistic insight^4^.

Mass spectrometry based quantification of proteins using lanthanides is a relatively new approach to observing multiple markers in single cells within tissues. We have previously used fluorescence imaging to good effect^6,14,15^ but need a more comprehensive view of the changes in cells as they are progressively affected by mitochondrial dysfunction. Single cell proteomics has already provided new insights, but this approach does not readily evaluate the heterogeneity of cell state in a diseased tissue where it is well known that there is particularly high levels of cell to cell variation. Here we aimed to evaluate this new approach and establish the advantages and limitations in the overall protocol before the method can be used for diagnostics or in drug screening. We demonstrate the use of this assay on ten patients with genetically confirmed mitochondrial disease due to pathogenic variants in either mtDNA or nDNA leading to a range of heterogeneous (mtDNA) or homogenous (nDNA) deficiencies of oxidative phosphorylation. Furthermore, we also developed an automated image segmentation software with an associated analysis pipeline that can be applied to the study of other heterogeneous pathological changes in skeletal muscle.

## Results

### IMC can be used to assess protein levels and cell morphology in skeletal muscle

We have optimised IMC for use in frozen skeletal muscle to determine the levels of metal-labelled antibodies targeting proteins of interest in a multiplexed assay at the single cell level. For this we targeted mitochondrial oxidative phosphorylation subunits in patients with mitochondrial disease as a particularly heterogeneous pathological phenotype. In addition to this, by single cell segmentation we can extract some relevant morphological measurements such as circularity, aspect ratio, convexity and area of fibre cross section as well as coordinates of fibre centres, which can be used for neighbourhood analysis.

### IMC produces comparable results to IF and is reproducible

**Figure 1:**
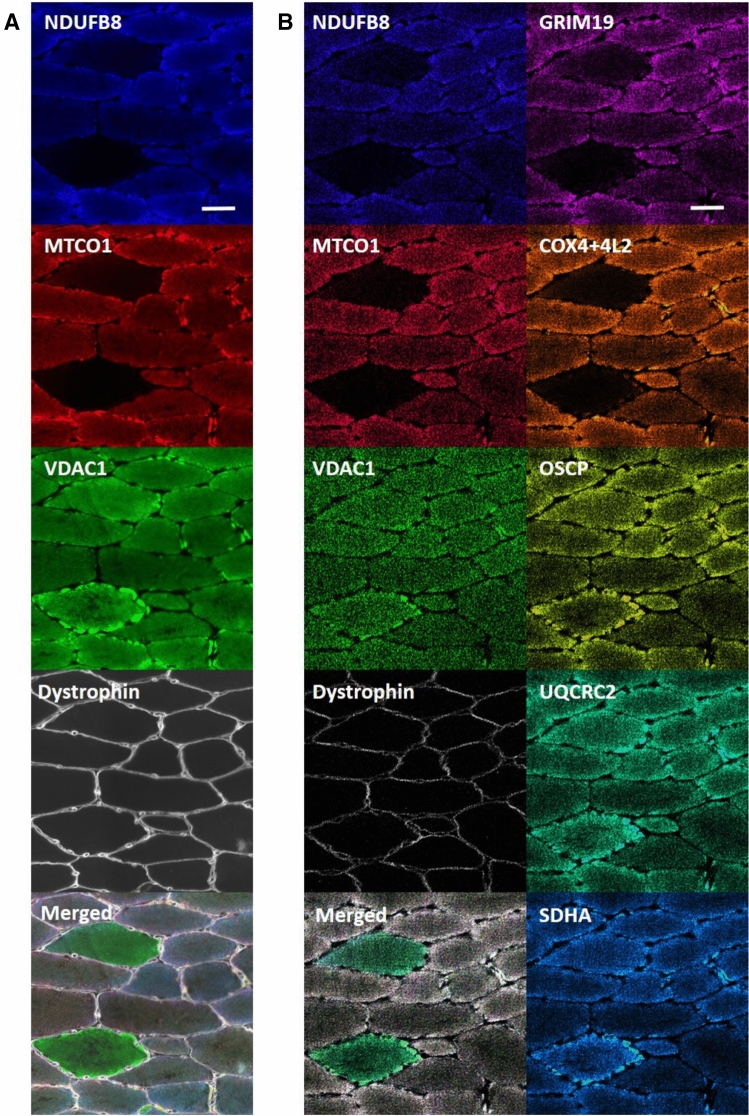
Labelling patterns for mitochondrial targets using immunofluorescence (IF) and imaging mass cytometry (IMC). Intensities scaled for each individual channel to fill bit-depth range for display and then merged. IF (**A**) and IMC (**B**) carried out on skeletal muscle tissue from P04 using antibodies recognising the indicated markers and an overlay of all analysed channels. Scale bar 50 μm. Capture resolution: 1 pixel/µm (IMC), 3.10 pixel/µm (IF).

To benchmark IMC against the previously validated IF assay^6^, we performed IF and IMC on serial sections to establish if quantification of changes in mitochondrial protein expression were equivalent between the two methods. By visual inspection, the images generated by both methods appeared to show similar staining patterns and similar subcellular distribution of all interrogated proteins (Fig. 1). For semi-quantitative analysis of protein levels using IMC and IF on sections from the same patients, we calculated the ratio of each subunit of oxidative phosphorylation relative to VDAC1 (a mitochondrial mass marker) for each fibre from each patient. In order to assess the relationship between the two methods, we then measured the linear correlation between these two ratios using Pearson’s correlation coefficient (calculated using the cor function in R). We found a strong correlation between log-transformed IMC data and IF data (Pearson’s correlation coefficient = 0.85).

To ensure that IMC results were reproducible, sections from two controls (C01 and C03) and two patients with mtDNA variants (P08 and P10 harbouring the m.10010T>C *MT-TG* and m.5543T>C *MT-TW* variants which caused the broadest range of oxidative phosphorylation deficiency) were stained and imaged on three separate occasions, once with one batch of antibody conjugations and twice with a new batch of antibody conjugations. Results were reproducible between samples analysed with the same reagents on different days for two serial muscle sections (Pearson’s correlation coefficient = 0.95). Each time a new vial of antibody is conjugated to a heavy metal the yield and concentration of the antibody may be different to previous or subsequent conjugations of the same antibody. We therefore looked to assess how important it is to ensure the same batch of antibody is used for comparisons. When we compared IMC experiments in which we used antibodies from different conjugation batches we found that, although still well correlated, there was greater variability between the two runs (Pearson’s correlation coefficient of 0.87 between original run and each replicate, respectively). This variability is likely caused by differences in antibody recovery and in the density of metal labelling of the antibodies.

### plotIMC permits interaction with IMC data

Since IMC permits the analysis of many more targets than IF^6^ and in order to deal with the increased complexity and heterogeneity of IMC data, we developed a novel, user-friendly, interactive web-tool which we refer to as plotIMC (http://mito.ncl.ac.uk/warren_2019).

When examining the expression of mitochondrial proteins, it is important to account for mitochondrial mass as previously described^6^. Here, plotIMC uses two related approaches to account for mitochondrial mass as each one highlights different characteristics of expression and biochemical deficiency. The first approach is visual inspection of 2Dmito plots (e.g. Web Figure p1). 2Dmito plots are scatterplots comparing cell-average IMC measurements for each antibody target observed during the IMC run with a surrogate for mitochondrial mass (VDAC1). The second approach is to examine the angle (theta) that each fibre in a 2Dmito plot makes with the origin (0,0) and the x-axis (e.g. Web Figure p2). Theta represents the level of expression relative to mitochondrial mass. In both views, each point represents a single fibre—grey points represent fibres from the controls and the patient points can be colour coded according to the expression of the proteins selected in the ‘colour fibres by channel’ drop down menu. In addition to this plot IMC can easily be used to look at mean intensity of each target protein without correction to a cellular or organelle mass marker as we use here (Web Figure p3). Using plot IMC we are able to pull out a range of interesting findings from this cohort of patients with mitochondrial diseases, which we present as an example of the possible applications.

### A spatial pattern in biochemical deficiency in some mitochondrial patients

**Figure 2:**
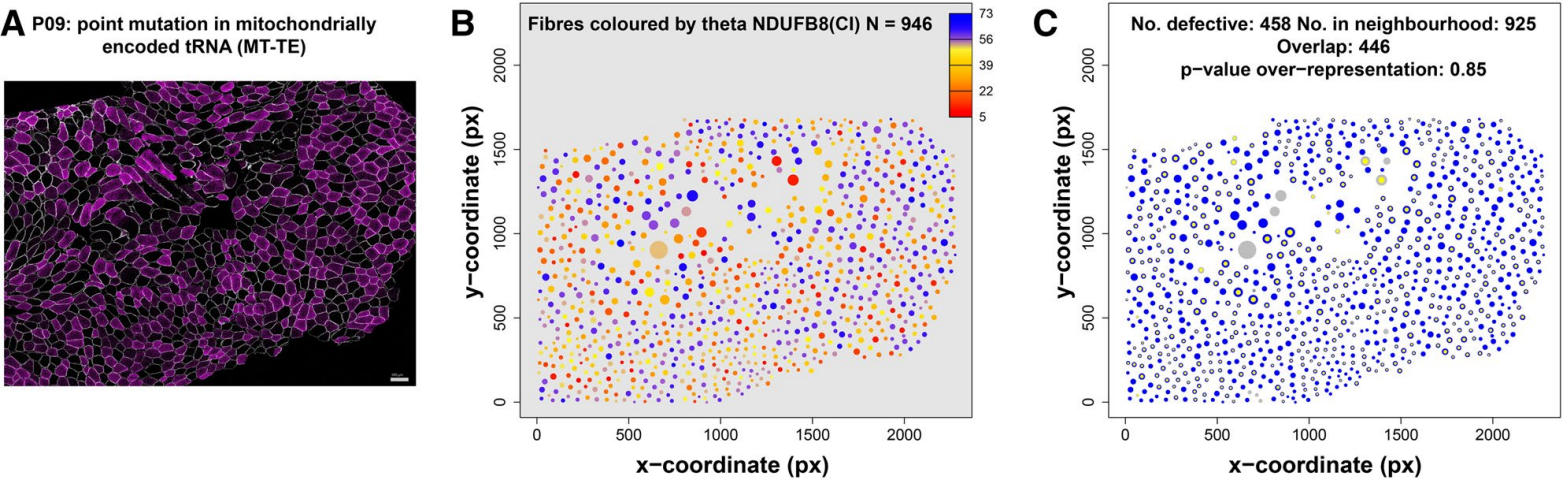
Spatial variation in biochemical deficiency in skeletal muscle biopsy cross-section taken from patient with a m.14709T>C *MT-TE* tRNA variant. (**A**) One of 9 raw pseudo-images from IMC from P09 [point mutation in mitochondrial-encoded tRNA (*MT-TE*)]. Dystrophin (a membrane-associated cytoskeletal protein) expression levels shown in grey, NDUFB8 in magenta. (**B**) Quantification of spatial pattern in NDUFB8 expression levels (theta) observed in (**A**). Continuous range of theta values represented by colour (orange fibres are deficient). (**C**) Representation of biochemically deficient fibres (yellow) and their neighbours (blue) after analysis of data from (A) (and VDAC1 data and comparison with controls). Fibre area in both panels represented by circle area.

In patients with mutations in mtDNA, there is a considerable amount of heterogeneity between the biochemical status of muscle fibres. Some of that variability could be attributed to spatial effects, such as the lineage of cells resulting from tissue development ^16^ or due to horizontal transfer of mutant mtDNA between adjacent cells, for example ^17,18^. We can use mitocyto to quantify and visualise spatial effects in skeletal muscle sections. Most of these patients show a random spatial pattern, an example from P09 (m.14709 T > C *MT-TE* variant) is presented in Fig. 2. The heterogeneity of protein expression in the patient sample (Fig. 2A) is quantified in Fig. 2B. Here, fibres that are biochemically normal (52%, theta ~ 45°—70°) and biochemically deficient (48%, theta < 40°) are shown by colour whilst the size of the dots correspond to the size of the fibre. This analysis has potential to identify other interesting spatial patterns in future cohorts of patients.

### A compensatory increase in complexes II-V is observed in patients with complex I assembly defects

**Figure 3:**
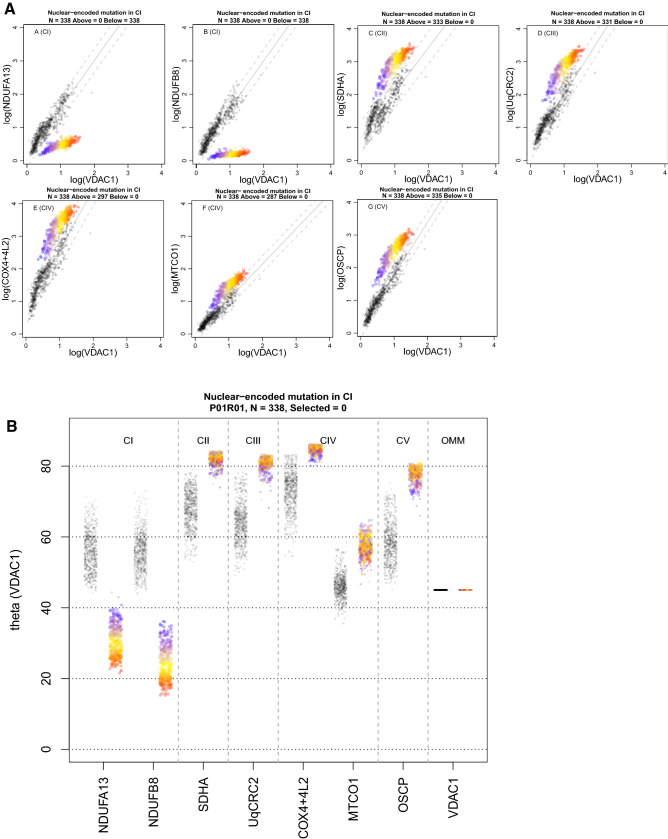
Imaging mass cytometry (IMC) results from P01 with a nuclear-encoded complex I (CI) pathogenic variant in *TMEM126B*. One single-cell IMC measurement from the patient is represented by a coloured point in each panel. Control observations are in grey. Each plot represents an antibody observed during the IMC run. (**A**) 2Dmito plot view presents each oxidative phosphorylation marker plotted against VDAC1. Points representing patient fibres are coloured by theta for NDUFB8 for that fibre. Regression through the control data is drawn as a solid grey line and the 95% predictive interval for control fibres lies between the dashed grey lines. Total number of fibres above and below the dashed lines are written above each panel. (**B**) Theta plot shows for each individual fibre the angle that is generated between the point and the x-axis on the 2Dmito plots. Theta quantifies expression of each oxidative phosphorylation marker in the context of mitochondrial mass (VDAC1). Points representing patient fibres are coloured for expression (theta) of NDUFB8; red fibres have the lowest expression and blue fibres highest expression. Web Figure: p1.

Patients 1 (P01) and 2 (P02) harbour pathogenic variants in the nuclear-encoded CI assembly factors TMEM126B and ACAD9, respectively (Supplementary Table S1). Ahmed et al.^15^ previously reported that 100% of fibres analysed were deficient for CI (based on NDUFB8) for both P01 and P02. As expected, we observed lower levels of CI proteins NDUFB8 and NDUFA13 relative to controls in both patients’ muscle fibres, with 100% of fibres for both P01 and P02 lying below the 95% predictive interval for the controls (example P01 in Fig. 3A). We found a strong correlation between the levels of NDUFB8 and NDUFA13 (Pearson’s correlation coefficient = 0.95 and 0.93 for P01 and P02, respectively), both complex I subunits.

In response to the deficiency in CI protein expression, we detected an upregulation of CII, CIII, CIV and CV proteins in muscle fibres in both P01 and P02; 98.5%, 97.9%, 84.9% and 99.1% of fibres lie above the 95% predictive interval for SDHA, UqCRC2, MTCO1 and OCSP respectively for P01. 70.2%, 71%, 88.9% and 61.5% of fibres lie above the 95% predictive interval for SDHA, UqCRC2, MTCO1 and OCSP respectively for P02. However, by colouring of data points for NDUFB8 (Fig. 3B), we can demonstrate that the downregulation in CI is not well correlated with the degree of upregulation in CII, III, IV and V.

**Figure 4:**
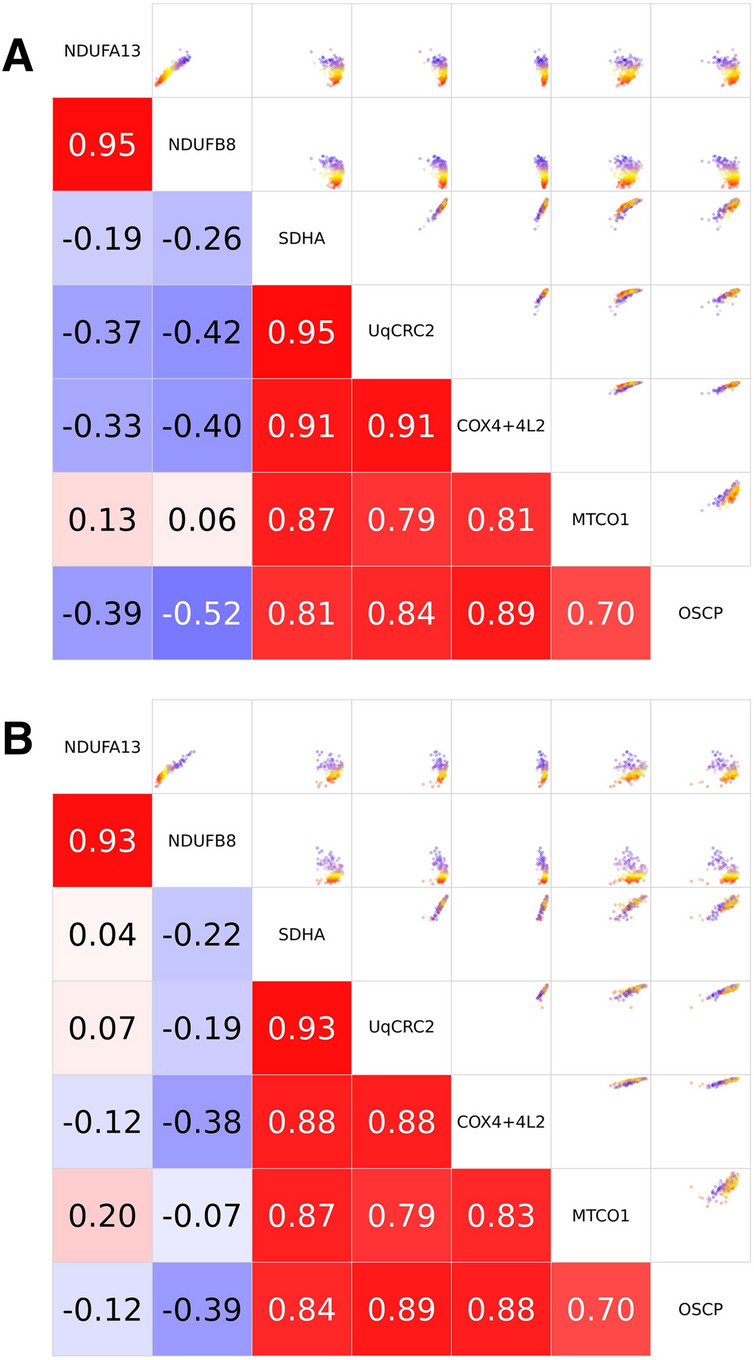
Imaging Mass Cytometry (IMC) results from P01 and P02 with a nuclear-encoded complex I (CI) variant. Correlation matrix. Scatterplots demonstrating correlation (upper right) and Pearson’s correlation coefficients (bottom left) between expression levels (theta) of each pair of proteins for all fibres. (**A**) P01 with pathogenic *TMEM126B* variants and (**B**) P02 with pathogenic *ACAD9* variants.

This is further evidenced in the correlation matrices and Pearson’s coefficents for patients P01 and P02 (Fig. 4). Both patients exhibit strong correlation coefficients for markers of CI, CIII, CIV and CV and for the two markers of CI, but weak correlations between CI markers NDUFA13 and NDUFB8 and markers of CII, CIII, CIV and CV.

### Oxidative phosphorylation protein levels vary between individual fibres from patients with pathogenic mtDNA variants

#### Single, large-scale mtDNA deletions

**Figure 5:**
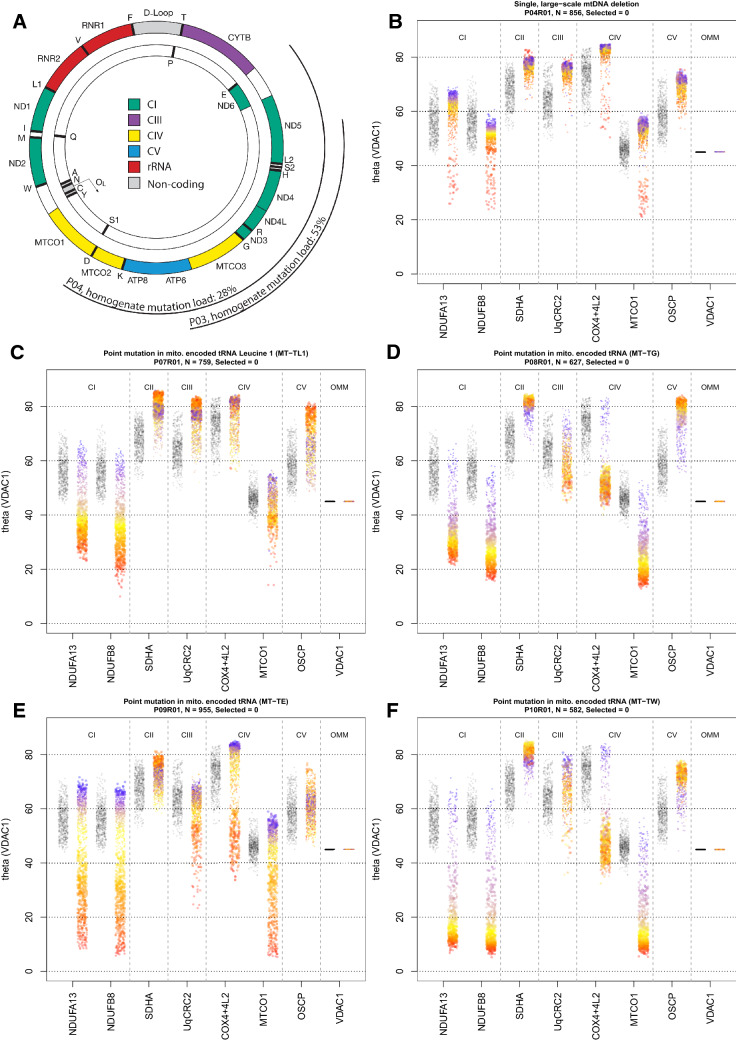
IMC results from one patient from each patient group. (**A**) Location and size of the mtDNA deletion from individual patients. P03 has a deletion of 4372 bp, with breakpoints: m.8929-13301, the deletion removes part of *ATP6, MTCO3, MT-TG, ND3, MT-TR, ND4L, ND4, MT-TH, MT-S2, MT-L2* and part of *ND5* and has a homogenate mutation load of 53%. P04 has a deletion of 7498 bp, with breakpoints: m.7130-14628, the deletion removes part of *MTCO1, MT-S1, MT-TD, MTCO2, MT-TK, ATP8, ATP6, MTCO3, MT-TG, ND3, MT-TR, ND4L, ND4, MT-TH, MT-S2, MT-LS, ND5, ND6, MT-TE* and part of *CYTB*, with a homogenate mutation load of 28%. The mtDNA is colour-coded by gene-type: CI genes; green, CIII genes; purple, CIV genes; yellow, CV genes; blue, rRNA genes; red and tRNA genes (black). Stripcharts showing theta values for all targets. Theta is the angle a single point makes with the x-axis and origin in a 2Dmito plot. (**B**) P04 harbouring a single, large-scale mtDNA mutation, (**C**) P07 with a mutation in m.3243A>G, (**D**) P08 with a point mutation in mitochondrial-encoded tRNA (m.10010T>C *MT-TG*), (**E**) P09 with a point mutation in mitochondrial-encoded tRNA (m.14709T>C *MT-TE*) and (**F**) P10 with a point mutation in mitochondrial-encoded tRNA (m.5543T>C *MT-TW*). One single-cell IMC measurement from the patient is represented by a coloured point in each panel. Control observations are in grey. Each pair of strips represents an antibody observed during the IMC run. Data presented using plotIMC theta (VDAC1) view. Points representing patient fibres are coloured for theta NDUFB8 for that fibre.

P03 and P04 both harbour single, large-scale heteroplasmic mtDNA deletions within the major arc of the mtDNA sequence, leading to the loss of numerous mt-mRNA and mt-tRNA genes (Fig. 5A; Supplementary Table S1).

P03 had a small proportion of deficient fibres with only 4.6% of the fibres below the 95% predictive interval for NDUFB8 and fewer fibres below the 95% predictive interval for NDUFA13 (2.4%), and MTCO1 (2.7%) (Web Figure p4). In addition, we also find 0.8% and 1.5% of fibres below the predictive interval for UqCRC2 and OSCP respectively.

P04 (Fig. 5B, Web Figure p5) has a clear subpopulation of fibres deficient in both CI and CIV subunits: 28% and 5% of fibres lie below the 95% predictive interval for CI subunits NDUFB8 & NDUFA13 respectively and 7% and 6% for CIV subunits MTCO1 and COX4 + 4L2 respectively, with the vast majority of CIV-deficient fibres also being deficient in CI protein expression. This is consistent with previous IF data acquired in muscle from the same patient^6^. In addition, we find 0.4% of fibres below the predictive interval for UqCRC2. We also observed that there were high levels of SDHA (theta > 80°) in the presence of severe defects in both CI and CIV (theta < 40°) but no CII upregulation with CIII or CV deficiency.

#### m.3243A > G mt-tRNA^Leu(UUR)^ (MT-TL1) variant

P05, P06 and P07 all harboured the m.3243A>G *MT-TL1* mtDNA variant which may affect the expression of subunits of CI and CIII–CV given these components of oxidative phosphorylation all contain mitochondrially-encoded proteins. Data from P07 are shown as an example in Fig. 5C and all data presented in Web Figures p6, p7 and p8. We observed 29.8%, 23.1% and 70.1% of fibres lying below the 95% predictive interval for NDUFB8 compared to 14.3%, 9.5% and 19.8% of fibres lying below the 95% predictive interval for MTCO1, in P05, P06 and P07 respectively (Web Figures p9, p10 and p11), which is consistent with previous studies assessing oxidative phosphorylation deficiency in muscle from patients with m.3243A>G variants^6^. We demonstrated that NDUFB8 is more deficient than NDUFA13, with a smaller proportion of fibres falling below the 95% predictive interval for NDUFA13 compared to NDUFB8 in all three patients (Web Figures p9, p10 and p11). All fibres with low levels of MTCO1 expression also demonstrated low NDUFB8 levels. In these patients, the muscle fibres which were most deficient in CI (theta < 40° for NDUFB8) demonstrated a compensatory increase in SDHA. Similarly, SDHA levels are higher than controls for CIV-deficient fibres suggesting a compensatory change in response to deficiency. Upregulation of UqCRC2 (43.8%, 55.4% and 66.7% for P05, P06 and P07) and OSCP (17.9%, 11.5% and 41.9% for P05, P06 and P07) was also observed in all m.3243A>G patients, though the degree of this compensatory response was not well correlated with the level of oxidative phosphorylation deficiency. This may, in part, be due to a low proportion of UqCRC2 deficient fibres in all patients (4.0%, 3.4% and 0.3% in P05, P06 and P07 respectively) and OSCP deficient fibres in two of the three patients (1.0% and 2.4% in P05 and P06, respectively). Those fibres with theta greater than controls for UqCRC2 and OSCP demonstrate CI or CI and CIV deficiency.

We find that P05 and P06 have fibres with high levels of VDAC1 expression that are likely ragged-red fibres (RRF; Web FIgures p9 and p10) but these fibres do not have a corresponding upregulation of SDHA relative to VDAC1. Contrastingly there are also a subset of fibres that have normal levels of VDAC1 which lie above the 95% predictive interval for SDHA, indicative of an overexpression of CII.

#### Other pathogenic mt-tRNA variants

P08, P09 and P10 all harboured pathogenic mitochondrial tRNA variants; P08 a m.10010T>C *MT-TG* variant (Fig. 5D, Web Figure p12), P09 a m.14709T>C *MT-TE* variant (Fig. 5E, Web Figure p13) and P10 a m.5543T>C *MT-TW* variant (Fig. 5F, Web Figure p14). All three variants can, in principle, affect the expression of mitochondrial-encoded subunits of CI and CIII–CV, however the effect they have on each of these complexes may differ and the detectable change will depend on the relative positions during oxidative phosphorylation complex assembly of the mtDNA encoded subunits and subunits targeted by the IMC assay. All three patients exhibited a proportion of muscle fibres deficient in both CI and CIV. 89.1%, 48.2% and 88.3% of fibres were found to lie below the 95% predictive interval for NDUFB8 and 89.3%, 35.1% and 85.9% of fibres lying below the 95% predictive interval for MTCO1, in P08, P09 and P10 respectively (Web Figures p15, p16 and p17). Unlike patients with other pathogenic mtDNA variants (single, large-scale mtDNA deletion and m.3243A>G *MT-TL1*), in these patients, the proportion of fibres deficient in CI is similar to the proportion deficient in CIV. In addition, NDUFB8 and NDUFA13 protein expression were equally affected in muscle fibres of P08–P10. Furthermore, we also observed an accompanying increase in both CII and CV in response to deficiency, with 89.5% and 58.1% of fibres above the 95% predictive interval for SDHA for P08 and P10 respectively and 90.75% of fibres above the 95% predictive interval for OSCP in P08. CIII-deficiency (UqCRC2) was present in a greater proportion of fibres than in other patients examined here, with 55.8%, 30.1% and 59.9% of fibres below the 95% predictive interval for P08, P09 and P10). In addition, 21.6% of fibres from P09 were below the 95% predictive interval for OSCP.

### The thresholds at which oxidative phosphorylation occurs are different for each complex

**Figure 6:**
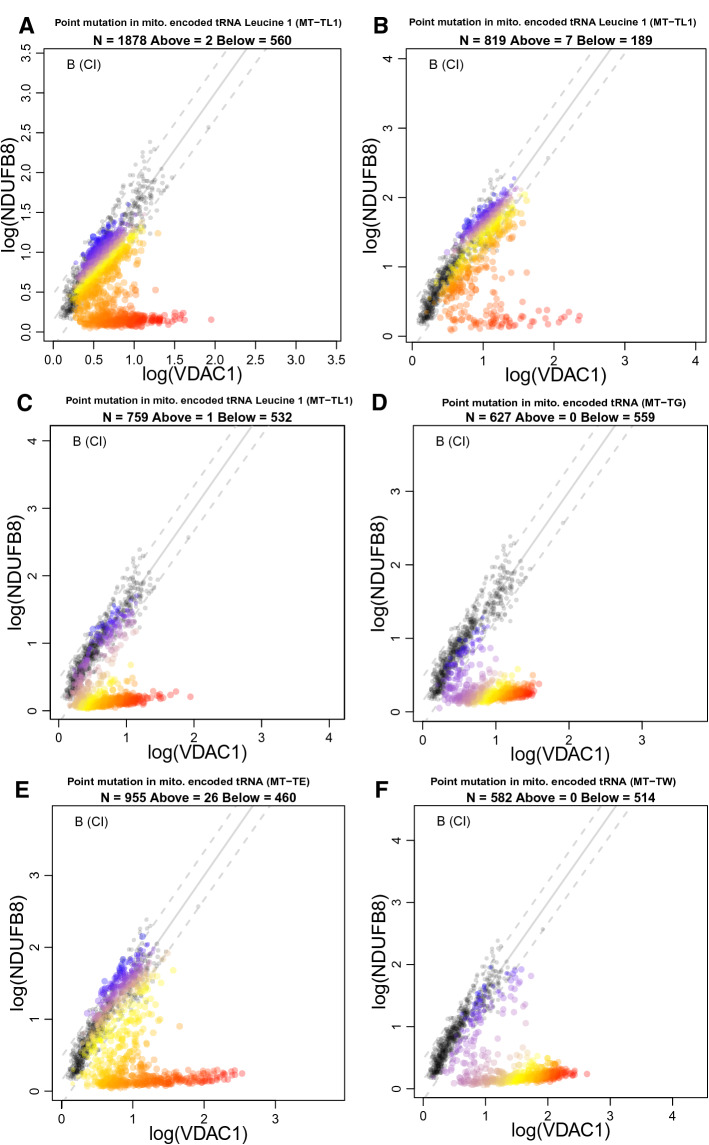
Muscle fibres in patients with mtDNA encoded tRNA variants typically fall into two distinct populations for NDUFB8 levels. 2Dmito plots comparing oxidative phosphorylation targets against VDAC1. (**A**) P05 with a mutation in m.3243 A>G, (**B**) P06 with a mutation in m.3243A>G, (**C**) P07 with a mutation in m.3243A>G, (**D**) P08 with a point mutation in mitochondrial-encoded tRNA (m.10010T>C *MT-TG*), (**E**) P09 with a point mutation in mitochondrial-encoded tRNA (m.14709T>C *MT-TE*) and (**F**) P10 with a point mutation in mitochondrial-encoded tRNA (m.5543T>C *MT-TW*). Individual single-cell IMC measurement from each patient is represented by a coloured point in each panel. Control observations are in grey. Points representing patient fibres are coloured for by theta NDUFB8 for that fibre. Regression through the control data is drawn as a solid grey line and the 95% predictive interval for control fibres lies between the dashed grey lines. Total number of fibres above and below the control predictive interval are written above each panel.

Multi-dimensional IMC data has the potential to be very useful to investigate pathogenic mechanisms in neuromuscular diseases. In all patients with pathogenic variants in mt-tRNA genes (P05–P10), we find that the fibres are split into two groups for CI markers. In the first group the relationship between CI protein levels and VDAC1 protein levels resembles control patients (e.g. patient fibres where the linear regression between NDUFB8 and VDAC has a similar gradient to that of the controls, as in Fig. 6A), in the second group the signal resembles patients with a nuclear-encoded CI mutation (e.g. patient fibres lying along the x-axis, with a regression line that has a gradient close to zero, as in Fig. 6A). This qualitative split into two populations, evident in the v-shaped phenotype that we show for the first time in Fig. 6, is consistent with previous studies where a threshold mtDNA mutation load must be achieved before cells show signs of biochemical deficiency. Rossignol and colleagues described a phenotypic threshold effect whereby a clinical manifestation only occurs when the proportion of wild type and mutated mtDNA is shifted in favour of a higher mutant load^19^. Further to this, it has previously been demonstrated that this threshold can vary for different complexes in single, large-scale mtDNA deletion patient muscle where CIV exhibits a higher threshold than CI^14^. In support of these findings, our data demonstrate that the proportion of CIV-deficient fibres appears to be smaller than the proportion of CI-deficient fibres in individual patients with pathogenic mtDNA variants. Furthermore, we demonstrate data consistent with a similar threshold sequence for CIII and CIV, where most CIII-deficient fibres are also deficient for CIV. Given that these cells have the same mtDNA mutation and that a higher mtDNA mutation load is associated with a greater degree of oxidative phosphorylation deficiency, this suggests that the threshold mtDNA mutation load for CIII deficiency is higher than that for CIV deficiency which in turn is higher than that for CI deficiency. In order to assess this, further investigation of the overlap between CI-, CIII- and CIV-deficient fibres coupled with single cell genetic analysis will be required.

## Discussion

In this work, we sought to evaluate the application of IMC to assess mitochondrial oxidative phosphorylation dysfunction in skeletal muscle. IMC has allowed us to analyse the levels of nine proteins simultaneously in over eight thousand single skeletal muscle fibres. We have validated the application of IMC to the study of skeletal muscle by observing strong correlation with protein expression levels estimated by IF. We have also built a novel, user-friendly, interactive web-tool that enables visualisation and analysis of data from individual muscle fibres and is suited to heterogeneous biopsy samples.

Not only does this work pave the way for systematic, single-cell observations of oxidative phosphorylation deficiency and response to deficiency in a clinically-relevant tissue, but also for other pathologically relevant protein and cell morphology changes. Such a technique will prove crucial in delineating the cellular changes and disease mechanisms associated with muscle ageing and neuromuscular disease. IMC also has potential as a high-throughput screening method and could be used in a diagnostic setting, the major limitations being the time to label and optimise antibodies and the accessibility of ICP-MS. How useful this would be for mitochondrial and other muscle diseases will depend on the success of current diagnostic algorithms.

Visualising and interpreting the large multi-dimensional datasets generated by IMC was particularly challenging. It was not possible to automatically classify patients with heteroplasmic mtDNA disorders from single cell IMC data, using standard multi-dimensional statistical techniques. This was likely due to heterogeneity of cells across individual muscle biopsies, which is also likely to be a problem for other heterogeneous pathogenic features in skeletal muscle. We therefore developed plotIMC, a web-tool which facilitates the interactive visualisation and browsing of IMC datasets, to examine oxidative phosphorylation protein status in any subset of thousands of cells simultaneously. plotIMC is designed specifically to highlight features that are most relevant for mitochondrial disease, such as a decrease or increase in components of oxidative phosphorylation relative to mitochondrial mass and an overall increase in mitochondrial mass as observed in oxidative phosphorylation-deficient ragged-red fibres, while being general enough to allow the user to interrogate these data.

An important consideration during the development of an IMC assay is the selection of protein targets with validated and specific antibodies, alongside cell markers for automated cell-segmentation. A determining factor in the inclusion of certain antibodies was whether they were available to purchase in a protein-free solution and compatibility of labelling protocols. Ideally, assessing fibre type distribution alongside mitochondrial antibodies would be a useful and interesting avenue to explore, however, fibre typing antibodies were not compatible with the protocol for antibody labelling of mitochondrial targets.

Whilst this oxidative phosphorylation IMC assay has applications in the study of mitochondrial dysfunction in ageing and disease, we chose to test our approach on muscle biopsies from patients with mitochondrial disease. We have been able to confirm previously published results demonstrating the validity of this technique compared to those used previously.

A proteomic investigation of single, large-scale deletion patients previously reported lower expression of nuclear and mtDNA encoded subunits of complex I and complex IV in COX-deficient fibres^13^, which is paralleled in our study of muscle from patients with single, large-scale mtDNA deletions. In addition, they observed decreased abundance of mtDNA-encoded subunits of complexes III and IV but no changes in nuclear-encoded oxidative phosphorylation subunits. In comparison, we detected a small percentage of muscle fibres with lower levels of nuclear-encoded CIII and CV subunits in a single, large-scale mtDNA deletion patient biopsy. Such a discordance could be explained by our study of individual muscle fibres compared to the pooling of COX-positive and COX-deficient fibres by Murgia et al.^13^. Whilst they did undertake single cell proteomic analyses, a sample of three for each group is unlikely to accurately represent the range of oxidative phosphorylation diversity we observe in our experiments. We observed similarly low levels of CIII deficiency in two out of three patients with the m.3243A>G *MT-TL1* variant and those with variants in with *MT-TG*, *MT-TE* and *MT-TW*. CV deficiency was further reported in two out of three patients with the m.3243A>G *MT-TL1* variant and patients with variants in *MT-TE* and *MT-TW*. CIII and CV deficiency occur in a smaller percentage of fibres than CI and CIV and are commonly found in fibres with CI and CIV deficiency, this is suggestive of a sequential genetic threshold hypothesis for the development of oxidative phosphorylation deficiencies, similar to that previously reported by Rocha et al.^14^. We have clearly demonstrated an increase in complexes of oxidative phosphorylation that are not directly affected by a mitochondrial or nuclear mutation occurs in fibres deficient in one or more oxidative phosphorylation complexes. Although previous biochemical studies report CI, CIII, CIV and CV upregulation with other isolated deficiencies^20,21^, here the pattern is reported in hundreds of single cells per patient. Most interestingly, using the ‘colour fibres by channel’ tool on plotIMC, we are able to demonstrate that patients with the m.3243A>G *MT-TL1* pathogenic variant show an upregulation of CII which is inversely proportional to the level of CI deficiency in 2 out of 3 patients. Moreover, this effect was not only observed in response to CI defects, but a similar pattern of CII protein upregulation is documented in fibres with combined CI and CIV protein deficiency.

The greatest advantage of IMC compared to previous immunofluorescent techniques is its ability to interrogate a larger number of markers simultaneously in a single tissue section. To analyse the nine proteins described in this manuscript by immunofluorescence would have required serial tissue sections and cross matching of serial sections which is difficult to automate. Furthermore, oxidative phosphorylation deficiency is known to vary along the length of a muscle fibre, meaning it may change between serial sections^22–24^. The experiments carried out in this study were focussed on the assessment of transverse muscle sections. However, IMC could also be used to evaluate longitudinal sections of muscle fibres. This would be valuable to investigate segmental respiratory chain deficiency since it has not been possible to examine all five OXPHOS complexes along the length of a muscle fibre.

The staining procedure is a straightforward protocol which has been adapted from an established quadruple immunofluorescent technique^6^, allowing researchers to move from immunofluorescence to IMC easily. However, antibody conjugations are necessary and time-consuming, with one person able to conjugate four antibodies in a day. The time for data acquisition per section is longer than conventional microscopy, however the imaging mass cytometer can run without manual intervention, imaging arrays of sections per slide. Furthermore, as IMC is more widely used and the panel of antibodies gets larger, the rate at which data that can be generated simultaneously and on the same tissue section, will far outweigh the running time of the machine. As with immunofluorescence, we automated segmentation of IMC images, making the two techniques equivalent in time taken for data extraction.

One potential difficulty when studying large cohorts is variability in antibody yield and concentration between conjugation batches. If such a large-scale experiment was necessary, a larger batch of antibody could be conjugated to cover the entire experiment.

In conclusion, we have optimised and developed an IMC assay for use on skeletal muscle cryosections, successfully validating this assay against an established quadruple immunofluorescent assay. We have demonstrated the suitability of IMC to analyse highly heterogeneous muscle biopsies from ageing and disease, by assessing mitochondrial oxidative phosphorylation dysfunction in genetically confirmed mitochondrial disease. Furthermore we have demonstrated the use of IMC for neighbourhood analysis which has potential for the study of diverse neuromuscular pathology. The development and future enhancement of IMC will open up many opportunities for deeper analyses of protein levels, cellular populations and cell morphology in single cells and sub-cellular regions to investigate the mechanisms of muscle ageing and neuromuscular pathology.

## Methods

### Cohort characteristics

Skeletal muscle biopsies were obtained from patients with clinically and genetically characterised mitochondrial disease with pathogenic mtDNA variants predicted to cause a generalised defect in mitochondrial protein synthesis (single, large-scale mtDNA deletion (n = 2), m.3243A>G *MT-TL1* (n = 3), m.10010T>C *MT-TG* (n = 1), m.14709T>C *MT-TE* (n = 1) and m.5543T>C *MT-TW* (n = 1)) and pathogenic nDNA variants in complex I (CI) assembly factors (n = 2). Ethical approval for use of mitochondrial disease patient tissue was granted by the Newcastle and North Tyneside Local Research Ethics Committee (reference 16NE/0267). Control tissue was acquired with prior informed consent from the distal part of the hamstring muscle of patients undergoing anterior cruciate ligament surgery, following approval by the Newcastle and North Tyneside Local Research Ethics Committee (reference 12/NE/0394). The experiments were carried out in accordance with the relevant guidelines and regulations. Information relating to the clinical presentation and variants found in the patients, together with a summary of control muscle samples, is presented in Supplementary Table S1.

### Antibodies and panel design

We designed a panel to include antibodies that targeted complexes I–V (CI–V), a membrane-associated cytoskeletal protein (dystrophin) and an outer mitochondrial membrane protein [Porin (VDAC1)]. A full list of antibodies used in this study can be found in Supplementary Table S2.

The antibodies to the mitochondrial proteins have been extensively validated and are widely accepted by the mitochondrial research community to be highly specific antibodies. They were originally developed by MitoSciences (now supplied by Abcam) and shown by the producers to selectively immunocapture their target protein/complex using mass spectrometry. Any non-specific labelling would have been detected by immunocapture and mass spectrometry. The antibodies have been extensively validated in knock out cell lines and are used routinely throughout the mitochondrial research community. Further, the reaction of the antibody in western blot, immunocytochemistry as well as IF correlates well with the genetic loss of protein as determined spectrophotometrically and by activity assays^25,26^. In addition, we have compared the antibodies pre- and post- conjugation with immunofluorescence to show that the conjugation process does not alter the staining pattern or binding affinity.

The antibodies against dystrophin and laminin have the characteristic staining pattern for the distribution of these proteins that is shown by multiple antibodies developed against these two proteins. Before conjugation, each antibody in the panel was ranked based on fluorescent intensity values from IF on control skeletal muscle and subsequently paired with an appropriate metal for conjugation ensuring that targets with a high fluorescent intensity were conjugated to a weaker metal and those with low fluorescent intensities with a stronger metal. Metal-labelled antibodies were conjugated using the Fluidigm protocol following manufacturer’s instructions. Antibodies were obtained in a carrier (BSA)-free buffer and prepared using MaxPar antibody conjugation kits (Fluidigm). As a final step, an antibody stabilisation solution was added before storage at 4 °C [Antibody stabiliser (PBS), Candor Bioscience]. After conjugation, the antibodies were incubated with antibody capture beads (AbC Total compensation beads, Thermo Fisher) and tested for the presence of the appropriate metal isotope signal by IMC in suspension mode (Helios). In order to check that the metal labelling process had not impaired the ability of each antibody to bind to the specified antigen the conjugated versions were then used to label control tissue followed by incubation with fluorescent secondary antibodies. These were compared to the pre-conjugated antibodies to confirm that the ranked levels were unchanged.

### Preparation of samples for imaging mass cytometry

Cryosections of optimal thickness for IMC (6 µm) were cut from transversely orientated cryoblocks and stored at −80 °C until required. The protocol used for antibody labelling is the same as reported previously^6^, with the exception of modifications stated below. The primary antibody cocktail was altered and is detailed in Supplementary Table S3. Since primary antibodies are conjugated to heavy metals this removes the need for incubation with secondary antibodies. All antibodies were used after a 1:50 dilution. Therefore, following the primary antibody incubation sections were washed for three times five minutes, before incubating with an Iridium (Ir) intercalator (Fluidigm), which labels DNA with a dual mass of 191/193 by time of flight (TOF) and thus acts as a nuclear marker, at a final concentration of 0.313 μM in TBST for 30 min at room temperature. Finally, the sections were washed in ddH_2_0 for 5 min before being air dried and placed in slide mailers ready to be taken to the imaging mass cytometer.

### Imaging mass cytometry

The hyperion tissue imaging module employs time-of-flight inductively coupled plasma mass spectrometry (ICP-MS) technology to measure the ions from each cell. IMC differs from conventional MS due to the introduction of a novel laser ablation device to the mass cytometer. The hyperion tissue imaging module was spatially aligned and coupled to the Helios mass cytometer prior to tuning the instrument at 20 Hz laser frequency with a standardised tuning slide and protocol (Fluidigm), which aims to bring signals within the optimum range. Resolution mass and optimal alignment was verified using the dual count of lutetium 175 and an acceptable signal cross talk at a 200 Hz laser frequency was verified. The instrument further measures a small number of channels for background, however due to the use of non-biological rare earth metals, background signal is minimal. Slide libraries were then generated using low resolution images to guide high resolution epiflourescent panoramas around selected regions of the tissue as guided by previous IF data. Region sizes (regions of interest, ROIs) were set to ablate sufficient numbers of fibres for downstream analyses based on a mean fibre density ~ 15 fibres per mm^2^ from 10 independent analyses. Prior to ablation, the optimal ablation energy was empirically determined in order to ensure that the full depth of the tissue was ablated. All ablations were performed according to a pre-generated mass template with a laser frequency of 200 Hz. After successful ablation, files were exported in their native “MCD” file format and then exported as single layer .TIFFs (16-bit) using MCD viewer software (Fluidigm).

### Preparation of samples for IF on serial sections

Serial 6 μm cryosections were subject to quadruple immunofluorescence as described previously^6^. Five different antibody combinations were used to allow for comparison of IMC and IF for all antibodies used in the IMC experiment in serial sections. Antibody combinations used on serial sections are detailed in Supplementary Table S4. No-primary antibody controls, incubated only with anti-laminin antibody followed by a full secondary antibody cocktail, were processed for each muscle sample to account for background signal.

### Imaging of sections prepared for immunofluorescence

Tiled images were acquired at a 20 × magnification using Zeiss Axio Imager M1 with a motorized stage and Zen 2011 (blue edition) software, with a monochrome Digital Camera (AxioCam MRm) and filter cubes for Alexa Fluor dyes at 405 nm, 488 nm, 546 nm and 647 nm wavelengths. Images generated by IF have a resolution of 3.10 pixels/μm. Optimum exposure times were set for all sections and maintained throughout. The tiles were then stitched and saved as .zvi files for further analysis.

### Analysis of immunofluorescence

IF analysis was carried out using an in-house Quadruple Immuno Analyser software (v3.6), as previously described^15^, which is now available at https://github.com/CnrLwlss/quad_immuno. The software segments muscle fibres based on membrane labelling and extracts single cell average fluorescent intensity used for downstream calculation of a range of statistics. All sections including the no primary controls were imaged at the same exposure time and fluorescent intensities of the no-primary control were quantified to determine levels of non-specific secondary antibody binding. Whilst background was low, we corrected for this on a patient by patient basis during quantitative analysis of protein levels, as previously described^6^.

### Analysis of IMC of tissue sections

Pseudoimages generated by IMC have a resolution of 1 pixel per 1 μm^2^, which is determined by the size of the laser spot. To segment and quantify IMC pseudoimages, we developed image analysis software (mitocyto (v.0.0.19): https://github.com/CnrLwlss/mitocyto) and applied it to our IMC data. Image stacks were segmented automatically using the dystrophin channel, or from a calculated gradient map of the mean of all channels, where possible. Identified areas thought to correspond to individual cells were filtered by size (areamin = 500 px, areamax = 17,500 px), circularity (circmin = 0.0, circmax = 100.0), aspect ratio (ratiomin = 0.0, ratiomax = 10.0) and convexity (convexmin = 0.75, convexmax = 1.0) to ensure that the area morphologies are such that they are likely, individual, transverse fibres sections. Segmentation was supplemented manually where signal was too weak for reliable identification of cell membranes. After segmentation, average signal intensity for each channel, total area, fibre coordinates and measures of morphology were reported in a text output file for subsequent analysis and visualisation.

### Visualising and interacting with IMC data

We summarised data from image analysis and attached metadata to data using the statistical programming language R (v4.0.0). plotIMC is a web-tool that we developed for interactive visualisation of multidimensional IMC data, using the R package shiny. A live instance of plotIMC, together with its documentation, can be found here: http://mito.ncl.ac.uk/warren_2019. Source code and raw data for plotIMC (v0.0.1), specific to this manuscript, together with some instructions for how to install and run plotIMC locally, can be found here: https://github.com/CnrLwlss/Warren_2019. Since IMC produces multiplexed single cell data similar to those from conventional flow cytometry techniques, we decided to analyse the data using standard multivariate statistical methods and carried out unsupervised k-means global clustering analysis of fibres from the full IMC dataset (results not reported). We found that the mixture of normal and deficient fibres in most of our patients rendered this clustering analysis very difficult to interpret and so developed an alternative approach based on interactive data exploration and analysis.

### Statistical analysis of IMC data

In order to account for the effect of mitochondrial mass, we fitted a linear regression model between values observed for each target antibody and VDAC1 in all control patients, noting the 95% predictive interval around that regression. The regression fit and predictive interval were generated using the lm function in R. Fibres are classified as being significantly affected by the background mutation of the patient if they lie outside the predictive interval for control fibres. This approach is used in the 2Dmito views in plotIMC.

In many cases a linear model is not a good fit to these data. As an alternative, we also classify fibres as oxidative phosphorylation-deficient by estimating theta, the angle each fibre makes with the origin and x-axis in a 2Dmitoplot. We generated a non-parametric kernel density estimate of the probability density function (PDF) for theta from data for controls and patient. We used the PDFs to estimate the likelihood of the theta value for each patient fibre coming from the control population and coming from the patient population. If the likelihood of coming from the patient population is higher than the likelihood of coming from for the control population (and lies outside the 95th percentile range for control fibres), that fibre was classified as affected. Kernel density estimates of PDFs were generated using the density function in R and using approxfun to interpolate between resulting density estimates. This approach was used to classify fibres as deficient in the stripchart views in plotIMC.

In order to calculate whether deficient fibres cluster together significantly we tested for over-representation of deficient fibres within the set of fibres that are neighbours to deficient fibres. A fibre’s neighbourhood was defined as any fibre where its centre lay within five times the average effective radius of fibres for that patient. Effective radius is defined as the radius of a circle with the same area as the fibre. Testing was carried out using the hypergeometric test (phyper function in R). Source code for analysis from this manuscript can be found here: https://github.com/CnrLwlss/Warren_2019.

## Supplementary information

Supplementary information
