# Supplementary material for: Decoding mitochondrial heterogeneity in single muscle fibres by imaging mass cytometry
Source: Sci Rep. 2020 Sep 18;10:15336. doi: 10.1038/s41598-020-70885-3 (PMC7501294; doi:10.1038/s41598-020-70885-3)
Supplement: Supplementary file 1 — Supplementary information [file 41598_2020_70885_MOESM1_ESM.pdf]

Decoding mitochondrial heterogeneity in single muscle fibres by Imaging Mass Cytometry

Charlotte Warren[1,4], David McDonald[2], Roderick Capaldi[3], David Deehan[4], Robert W. Taylor[1,5], Andrew Filby[2], Doug M. Turnbull[1,4], Conor Lawless[1,*], Amy E. Vincent[1,4*]

[1] Wellcome Centre for Mitochondrial Research, Translational and Clinical Research Institute, Newcastle University, Newcastle upon Tyne, NE2 4HH, United Kingdom.

[2] Flow Cytometry Core Facility, Biosciences Institute, Newcastle University, Newcastle upon Tyne, NE2 4HH, United Kingdom

[3] Cellstate Biosciences, Tuscon, Arizona 85721, United States

[4] Translational and clinical Research Institute, Newcastle University, Newcastle upon Tyne, NE2 4HH, United Kingdom.

[5]NHS Highly Specialised Rare Mitochondrial Disorders Service, Newcastle upon Tyne Hospitals, NHS Foundation Trust, Newcastle upon Tyne, NE2 4HH, United Kingdom.

*Corresponding author

Correspondence:

Dr Amy E. Vincent (email: amy.vincent@ncl.ac.uk)

Dr Conor Lawless (email: conor.lawless@ncl.ac.uk)

Wellcome Centre for Mitochondrial Research

Translational and Clinical Research Institute Newcastle University

Framlington Place,

Newcastle upon Tyne

NE2 4HH, UK

***Supplementary Table 1*: *Patient information*.** *Information on patients detailing gender, age at biopsy, clinical information and genetic defect.*

Subjects	Gender	Age (years)	Clinical information	Genetic defect	Previous reports
**Pathogenic variants in nuclear-encoded proteins affecting complex I assembly**					
P01	M	Adult	Exercise intolerance, unable to perform sustained aerobic exercise normal resting lactate, normal CK	*TMEM126B* (NG_053120.1)<br>Homozygous c.635G>T, p.(Gly212Val) variant	Ahmed *et al.* (2017) Patient 14
P02	M	Adult	Exercise intolerance, muscle cramps, elevated serum lactate	*ACAD9* (NG_017064.1)<br>Compound heterozygous c.1150G>A, p.(Val384Met) and c.1168G>A, p.(Ala390Thr) variant	Ahmed *et al.* (2017) Patient 15
**Single, large-scale mtDNA deletions**					
P03	F	29	CPEO and bilateral ptosis	Deletion size 4372bp<br>Breakpoints: 8929-13301<br>mtDNA deletion level: 53%<br>Deleted genes: part of *ATP6*, *MTCO3*, *MT-TG*, *ND3*, *MT-TR*, *ND4L*, *ND4*, *MT-TH*, *MT-S2*, *MT-L2* and part of *ND5*	Rocha *et al.* (2018) Patient 4
P04	F	39	CPEO, diplopia	Deletion size 7498bp<br>Breakpoints: 7130-14628<br>mtDNA deletion level: 28%<br>Deleted genes: part of *MTCO1*, *MT-S1*, *MT-TD*, *MTCO2*, *MT-TK*, ATP8, *ATP6*, *MTCO3*, *MT-TG*, *ND3*, *MT-TR*, *ND4L*, *ND4*, *MT-TH*, *MT-S2*, *MT-LS*, *ND5*, *ND6*, *MT-TE* and part of *CYTB*	Rocha *et al*. (2018) Patient 19
**Pathogenic variants in mitochondrially-encoded tRNA$^{Leu(UUR)}$ (*MT-TL1*)**					

P05	F	25	Exercise intolerance, ptosis	m.3243A>G *MT-TL1* variant	Rocha *et al.* (2015) Patient 12
P06	F	47	Modest exercise intolerance	m.3243A>G *MT-TL1* variant	Rocha *et al.* (2015) Patient 15
P07	M	53	CPEO	m.3243A>G *MT-TL1* variant	Rocha *et al.* (2015) Patient 16
**Point mutations in other mitochondrially-encoded tRNAs**					
P08	M	33	Mitochondrial myopathy	m.10010T>C *MT-TG* variant	Rocha *et al.* (2015) Patient 19
P09	F	35	Mild muscle weakness	m.14709T>C *MT-TE* variant	Rocha *et al.* (2015) Patient 20
P10	M	63	Exercise intolerance, prominent exertional dyspnea	m.5543T>C *MT-TW* variant	Rocha *et al.* (2015) Patient 21
**Healthy controls**					
C01	M	20	Taken during anterior cruciate ligament surgery	n.a	
C02	M	24	Taken during anterior cruciate ligament surgery	n.a	
C03	F	23	Taken during anterior cruciate ligament surgery	n.a	

CK; Creatine kinase, CPEO; Chronic progressive external ophthalmoplegia, n.a., not applicable

Supplementary Table 2: List of primary and secondary antibodies. *Table includes details of antibodies such as host species, isotype, dilution used and supplier.*

Antibodies	Host and isotype	Dilution	Company
**Primary antibodies**			
Laminin (membrane marker)	Rabbit	1:50	Sigma-Aldrich (L9393)
Dystrophin (membrane marker)	Mouse	1:50	EMD Millipore (Mab 1645)
NDUFB8 (CI)	Mouse IgG1	1:50 (IMC) 1:100 (IF)	Abcam (110242)
GRIM19 (CI)	Mouse IgG2b	1:50 (IMC) 1:100 (IF)	Abcam (110240)
SDHA (CII)	Mouse IgG1	1:50 (IMC) 1:100 (IF)	Abcam (14715)
UqCRC2 (C III)	Mouse IgG1	1:50 (IMC) 1:100 (IF)	Abcam (14745)
MTCO1 (CIV)	Mouse IgG2a	1:50 (IMC) 1:100 (IF)	Abcam (14705)
COX4+4L2 (CIV)	Mouse IgG2a	1:50 (IMC) 1:100 (IF)	Abcam (110261)
OSCP CV)	Mouse IgG1	1:50 (IMC) 1:100 (IF)	Abcam (110276)
VDAC1 (mass marker)	Mouse IgG2b	1:50 (IMC) 1:100 (IF)	Abcam (14734)
**Secondary antibodies**			
Anti-rabbit Alexa Fluor 405nm	Goat	1:100	Life Technologies (A31556)
Anti-IgG2a Alexa Fluor 488nm	Goat	1:200	Life Technologies (A21131)
Anti-IgG2b Alexa Fluor 546nm	Goat	1:200	Life Technologies (A21143)
Anti-IgG1 biotin	Goat	1:200	Life Technologies (A10519)
Streptavidin Alexa Fluor 647nm	Goat	1:100	Life Technologies (S32357)
Anti-IgG1 Alexa Fluor 647nm	Goat	1:200	Life Technologies (AA21240)
Anti-mouse IgG Alexa Fluor 488	Goat	1:200	Life Technologies (A11001)

Supplementary Table 3**: **Antibodies and their respective metal conjugates.

Antibody	Metal conjugate
Dystrophin	176Yb
UqCRC2	174Yb
MTCO1	172Yb
COX4+4L2	168Er
SDHA	153Eu
VDAC1	166Er
GRIM19	164Dy
OSCP	161Dy
NDUFB8	160Gd

Supplementary Table 4: Antibody combinations for comparison of Imaging mass cytometry and immunofluorescences*. Combinations of antibodies for quadruple immunofluorescence.*

**Combination 1**	**Combination 2**	**Combination 3**	**Combination 4**	**Combination 5**
Laminin	Laminin	Laminin	Laminin	Laminin
NDUFB8	NDUFB8	SDHA	OSCP	UqCRC2
MTCO1	COX4+4L2	TOMM22	MTCO1	MTCO1
VDAC1	VDAC1	GRIM19	VDAC1	VDAC1

Ahmed, S. T., Alston, C. L., Hopton, S., He, L., Hargreaves, I. P., Falkous, G., . . . Taylor, R. W. (2017). Using a quantitative quadruple immunofluorescent assay to diagnose isolated mitochondrial Complex I deficiency. *Scientific reports, 7*(1), 15676-15676. doi:10.1038/s41598-017-14623-2

Rocha, M. C., Grady, J. P., Grünewald, A., Vincent, A., Dobson, P. F., Taylor, R. W., . . . Rygiel, K. A. (2015). A novel immunofluorescent assay to investigate oxidative phosphorylation deficiency in mitochondrial myopathy: understanding mechanisms and improving diagnosis. *Scientific reports, 5*(1), 15037. doi:10.1038/srep15037

Rocha, M. C., Rosa, H. S., Grady, J. P., Blakely, E. L., He, L., Romain, N., . . . Turnbull, D. M. (2018). Pathological mechanisms underlying single large-scale mitochondrial DNA deletions. *Ann Neurol, 83*(1), 115-130. doi:10.1002/ana.25127
